# Anti-HIV Activity of Human Defensin 5 in Primary CD4+ T Cells under Serum-Deprived Conditions Is a Consequence of Defensin-Mediated Cytotoxicity

**DOI:** 10.1371/journal.pone.0076038

**Published:** 2013-09-25

**Authors:** Jian Ding, Carley Tasker, Kimyata Valere, Tiina Sihvonen, Dante B. Descalzi-Montoya, Wuyuan Lu, Theresa L. Chang

**Affiliations:** 1 Public Health Research Institute, Rutgers Biomedical and Health Sciences-New Jersey Medical School, Newark, New Jersey, United States of America; 2 Department of Microbiology and Molecular Genetics, Rutgers Biomedical and Health Sciences-New Jersey Medical School, Newark, New Jersey, United States of America; 3 Department of Pathology and Laboratory Medicine, Rutgers Biomedical and Health Sciences-New Jersey Medical School, Newark, New Jersey, United States of America; 4 Graduate School of Biomedical Sciences, Rutgers Biomedical and Health Sciences-New Jersey Medical School, Newark, New Jersey, United States of America; 5 Institute of Human Virology and Department of Biochemistry and Molecular Biology, University of Maryland School of Medicine, Baltimore, Maryland, United States of America; University of Iowa Carver College of Medicine, United States of America

## Abstract

**Background:**

We have previously shown that human defensin 5 (HD5) promotes HIV infectivity in both primary CD4+ T cells and HeLa cells expressing CD4 and CCR5. HD5 is induced in response to sexually transmitted infections (STIs) such as *Chlamydia trachomatis* and *Neisseria gonorrhoeae*, suggesting it plays a role in STI-mediated enhancement of HIV transmission. In contrast to our findings, a recent study reports that HD5 has an anti-HIV effect in primary CD4+ T cells under serum-deprived conditions. To resolve these apparently contradictory observations, we investigated experimental parameters that might contribute to contrasting effects of HD5.

**Results:**

Serum-deprived culture conditions were associated with anti-HIV activity. In contrast to the dependence of the HIV enhancing effect on HD5 structure, the anti-HIV activity in serum-deprived primary CD4+ T cells was independent of HD5 structure as the linear peptide [Abu] HD5 exhibited similar anti-HIV activity. Under serum deprived conditions, HD5 blocked CD4-receptor-independent HIV-1_vsv_ infection before or after viral entry. We found that HD5 and its linear form induced significant cell death in primary CD4+ T cells under serum-deprived culture conditions. HD5-mediated apoptosis was observed as early as 2 h after addition of defensins to serum-deprived primary CD4+ T cells. In contrast to primary CD4+ T cells, HD5 did not induce cytotoxicity and promote HIV infectivity of HeLa-CD4-CCR5 cells under serum-deprived conditions.

**Conclusions:**

These results indicate that under serum-deprived culture conditions HD5 is toxic for primary CD4+ T cells, warranting caution in data interpretation.

## Introduction

Defensins are cationic peptides that exhibit antimicrobial properties important to mucosal immunity (see reviews by Ganz, Salzman, and Lehrer and Lu [1], [2], [3]). It was initially thought that defensins inhibited primarily enveloped virus by disrupting the envelope membrane in a manner similar to their antibacterial activities [4]. However, recent progress in anti-viral activities of defensins has demonstrated that defensins block infection by both enveloped and non-enveloped viruses, and the precise effect of defensins on viral infection is specific to the triad of defensin, virus, and target cell (reviewed by Ding et al [5]). Additionally, defensins can promote or inhibit viral infection by modulating immune responses in animal models [6], [7].

Human defensin 5 (HD5) is the most abundant antimicrobial peptide in the intestine [8], a major site of CD4+ T cell depletion during acute HIV infection [9]. HD5 is constitutively expressed in Paneth cells in small intestine [10], and is also found in the female genital mucosa [11]. HD5 is found in genital fluid from individuals with *C. trachomatis* (CT) and *N. gonorrhoeae* (GC) infection and bacterial vaginosis (BV) [12], [13], and contributes to the enhancement of HIV infectivity provided by conditioned media from GC-exposed vaginal epithelial cells [14]. The HIV enhancing effect of HD5 is observed in infection of primary activated CD4+ T cells by both R5 and X4 primary isolates when viruses are pre-incubated with defensins in the absence of serum [14]. The enhancing effect is more pronounced with R5 virus than X4 virus [14]. This finding may have clinical implications as R5 virus is preferentially transmitted during primary infection [15], [16]. Further investigation of the underlying mechanism of HD5-mediated HIV enhancement demonstrated that HD5 promotes HIV attachment by concentrating virus particles on the target cells [17].

In contrast to these results from our laboratory [14], [17], a recent study reported contradictory results, showing that HD5 inhibited HIV infection of primary CD4+ T cells under serum-deprived conditions (0.3% human AB serum, ITS supplement (Insulin, Transferrin, Sodium selenite), and IL-2), conditions which the authors thought simulated the mucosal environment [18]. There was no preferential HIV effect on X4 or R5 virus [18].

In the current study, we sought to resolve this rather remarkable discrepancy and to understand the cause for the contrasting effect of HD5 on HIV replication in primary CD4+ T cells. In addition to differences in culture conditions of primary CD4+ T cells, the methods for CD4+ T cell isolation and virus inoculation also differed from our studies [14], [17]. We found that these latter differences in procedure also contributed to the discrepancy. We traced the mechanism of the anti-HIV activity of HD5 under serum-deprived conditions to defensin-mediated cell death, which is not known to occur in the milieu of the genital mucosa. Since abundant and diverse proteins are present in cervico-vaginal fluid [19], [20], [21] and lymphocytes are viable at the genital mucosa despite the enrichment of antimicrobial peptides including HD5 [1], [11], [22], [23], primary CD4+ T cells cultured under serum deprived conditions are unlikely to represent mucosal CD4+ T cells.

## Materials and Methods

### Reagents

Recombinant human IL-2 was purchased from R&D Systems (Minneapolis, MN). Histopaque®-1077, Triton X-100, RPMI-1640 medium, fetal bovine serum (FBS), human AB serum, ITS liquid media supplement (100X), and phytohemagglutinin (PHA) were from Sigma-Aldrich (St. Louis, MO). PerCP-conjugated mouse anti-human CD4 (clone RPA-T4) was from Biolegend (San Diego, CA). PE-conjugated mouse anti-human CD3 (clone UCHT1) and FITC Annexin V apoptosis detection kit I were from BD Biosciences (San Jose, CA). HD5 and its linear unstructured form, [Abu]HD5, in which the six cysteine residues were replaced by isosteric α-aminobutyric acid (Abu) were chemically synthesized and folded as described previously [24].

### CD4+ T Cell isolation

PBMCs from anonymous healthy blood donors from New Jersey Blood Center were used so the IRB approval was not required for this study. PBMCs were isolated by Histopaque®-1077 gradient centrifugation. Peripheral blood lymphocytes (PBLs) were obtained after removing monocytes by attachment. CD4+ T cells were isolated form PBLs by negative selection using a CD4+ T cell isolation kit II (Miltenyi, CA). Isolated CD4+ T cells were activated with 5 μg/mL PHA and 50 IU/mL IL-2 for 3 days (PHA-activated CD4+ T cells). Alternatively, PBLs were activated with 5 μg/mL PHA and 50 IU/mL IL-2 for 3 days. After washing with PBS 4 times, CD4+ T cells were isolated from PHA-activated PBLs by negative selection using the CD4+ T cell isolation kit II (CD4+ T cells from PHA-activated PBL) as described by Furci et al [18]. Cells were then cultured in the presence of 10%FBS and IL-2 or under serum-deprived conditions in the presence of 0.3% human AB serum, ITS supplement (Insulin, Transferrin, Sodium selenite), and IL-2.

### FACS analysis

The purity of CD4+ T cells prepared by different methods was analyzed by flow cytometry. Cells were first blocked with 2% FBS in PBS for 30 min on ice and then surface stained with fluorochrome-conjugated anti-CD3 and anti-CD4 Abs or isotype-matched control Abs on ice for 30 min. After washing with 2% FBS in PBS, cells were fixed with 2% paraformaldehyde in PBS for 20 min at room temperature. Surface expression of CD3 and CD4 were then analyzed on a BD LSR II. Twenty thousand cells were acquired per sample. Results were analyzed using FlowJo (Tree Star, OR).

To determine HD5-mediated apoptosis and cell death by flow cytometry, PHA-activated CD4+ T cells under serum-deprived conditions were treated with HD5 at different concentrations for 4 h or 24 h before staining with FITC Annexin V Apoptosis Detection Kit I per manufacture's suggestion.

### Cytotoxicity of HD5 and [Abu] HD5

PHA-activated CD4+ T cells or CD4+ T cells from PHA-activated PBLs (1×10^4^ cells per sample) were exposed to HD5 or a linear peptide [Abu]HD5 at various concentrations in serum-free (SF) RPMI-1640 medium at 37°C for 2 h or were centrifuged at 1250×g for 1.5 h. Cells were then plated in 96-well plates in RPMI containing 10% FBS and IL-2 or RPMI containing 0.3% human AB serum, 1× ITS supplement, and IL-2 for 24 h at 37°C. HD5 or [Abu]HD5 was present during the culture period. Cell proliferation was examined by MTS assay (Promega, CellTiter96 Aqueous One cell proliferation assay). Cytotoxicity was determined using a CytoTox-Glo cytotoxicity kit (Promega) following the manufacture's instruction. Briefly, 50 μL of assay reagent was added to each well and incubated for 15 min at room temperature. Dead cell luminescence (L1) was measured on a HARTA MicroLumi L2 luminometer (Gaithersburg, MD). Lysis reagent (50 μL) was then added to each well and incubated for another 15 min at room temperature. Total luminescence (L2) was then measured. Live cell luminescence was calculated as L2 minus L1.

### HIV-1 infection

HIV-1 primary isolate 20635–4 (R5 virus, clade C) [25] was obtained through the AIDS Research and Reference Reagent Program, Division of AIDS, NIAID, NIH from Dr. Smita Kulkarni. HIV-1 primary isolate was diluted in SF media to a final p24 concentration of 5 ng/ml in the presence of 1% FBS before incubation with defensins at different concentrations for 1 h at 37°C. The virus-defensin mixture was added to CD4+ T cells (2×10^5^ cells per sample). Spinoculation was performed by centrifugation at 1250×g for 1.5 h at room temperature. Samples without spinoculation were incubated at 37°C for 2 h. After washing off unbound virus, infected cells were cultured either in RPMI containing 10%FBS and IL-2 or under serum-deprived conditions (RPMI containing 0.3% human AB serum, 1× ITS supplement, and IL-2) in the presence of defensins. HIV-1 replication was determined by measuring the p24 level in the culture at day 6 of infection using a p24 ELISA kit from Advanced Bioscience Laboratories (Rockville, MD).

Replication-defective luciferase expressing reporter virus pseudotyped with R5 HIV-1_JR-FL_ Env or VSV-G for a single-cycle infection assay was produced in HEK293T cells as described previously [26], [27], [28]. After transfection, culture medium was changed to medium without serum 24 h before virus was harvested. Virus was incubated with various concentrations of HD5 or [Abu]HD5 at 37°C for 1 h before adding to cells. CD4+ T cells (5×10^5^ per sample) were incubated the virus-defensin mixture with or without spinoculation. After washing off unbound virus, infected cells were cultured for 3 days. Cells were lysed using passive lysis buffer (Promega, Madison WI) and luciferase activity (in relative light units [RLUs]) was measured on a Glomax 20/20 luminometer (Promega).

### Statistics analysis

Differences between data sets were analyzed by two-tailed Student *t* test. P<0.05 was considered significant.

## Results

### CD4+ T cells isolated from PHA-activated PBLs by negative selection are less pure than cells prepared from freshly isolated PBLs

**Figure 1:**
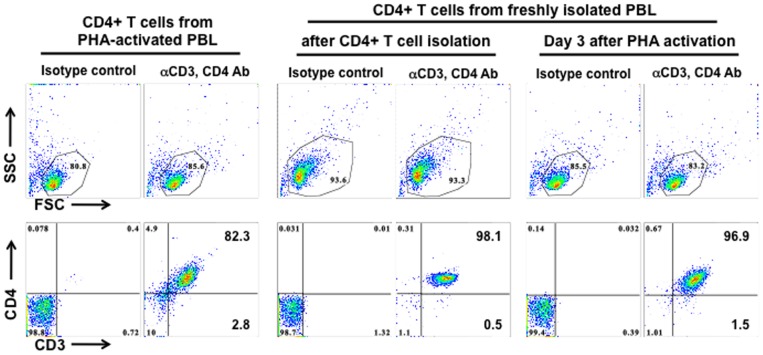
CD4+ T cells from PHA-activated PBLs are less pure than cells prepared from freshly isolated PBLs. CD4+ T cells isolated from PHA-activated PBLs (left panel), CD4+ T cells right after isolation from fresh PBLs (middle panel), or CD4+ T cells isolated from fresh PBLs followed by PHA activation for 3 days (right panel) were stained with PE-conjugated mouse anti-CD3 Ab and PerCP-conjugated mouse anti-CD4 Ab. Gated live cells in the scatter plot are shown in the upper panels. The results shown are representative of 3 tested donors, which are summarized in Table 1.

Several noticeable differences in the experimental system including the isolation of primary CD4+ T cells, virus infection method (spinoculation), and cell culture conditions may contribute to the contrasting effects of HD5 on HIV infection of primary CD4+ T cells observed by Furci et al [18] and by us [14], [17]. Furci et al activate PBMCs by phytohemagglutinin (PHA) followed by negatively selecting CD4+ T cells; we negatively select CD4+ T cells from freshly isolated PBMCs followed by activation of CD4+ T cells. We first compared the purity of CD4+ T cells between these two methods, and the results from three donors are summarized in Table 1. Analysis of CD4+ T cells negatively selected from PHA-activated PBL (CD4+ T cells from PHA-activated PBLs) revealed that approximately 80–85% of cells were viable (Fig. 1), 82.3% of which were CD3+CD4+ T cells. Other cell populations including CD8+ T cells (∼2.8%) were present. Analysis of CD4+ T cells using our method indicated that there were more than 93% viable cells, of which more than 98% were CD3+CD4+ T cells. There were approximately 83–85% live cells after PHA activation for 3 days (PHA-activated CD4+ T cells) but the purity of CD4+ T cells was greater than 96%, which was much higher than obtained by the method by Furci et al [18]. A small percentage of dead cells was expected, as activated human T cells release Fas (CD95) ligand (FasL) and APO2 ligand (APO2L)/TNF-related apoptosis-inducing ligand (TRAIL) leading to apoptosis [29].

**Table 1:** Purity of CD4+ T cells from different isolation protocols.

		CD4+ T cells from PHA-activated PBLs (percent)	PHA-activated CD4+ T cells (percent)	
			1 day	3 days
**Donor 1**	Viable	45.2	97.3	75.0
	CD3+CD4+	84.8	98.2	97.1
	CD3+CD4−	2.5	0.2	1.4
**Donor 2**	Viable	86.7	97.5	85.5
	CD3+CD4+	83.5	98.0	92.3
	CD3+CD4−	9.4	0.2	5.2
**Donor 3**	Viable	85.6	95.9	83.2
	CD3+CD4+	82.3	98.1	96.9
	CD3+CD4−	2.8	0.5	1.5

### Serum deprivation but not spinoculation contributes to anti-HIV activity of HD5 in primary CD4+ T cells

**Figure 2:**
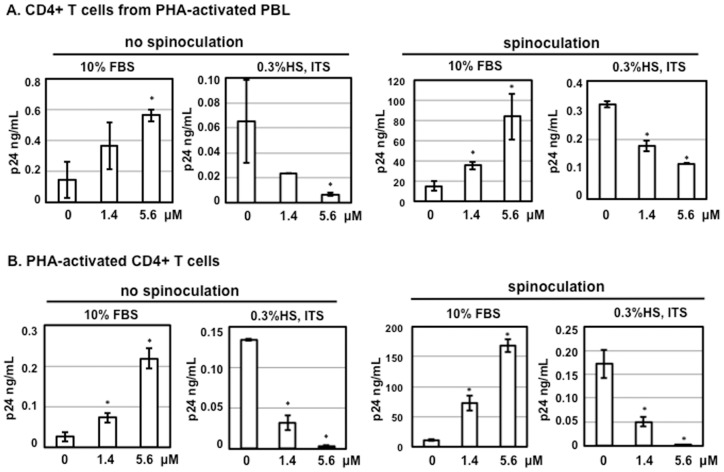
Serum deprivation contributes to anti-HIV activity of HD5 in primary CD4+ T cells. HIV-1 primary isolate 20635–4 (R5 virus, clade C) was incubated with HD5 at different concentrations for 1 h at 37°C. CD4+ T cells from PHA-activated PBLs (A) and PHA-activated CD4+ T cells (B) were incubated with virus-defensin mixture for 2 h at 37°C (no spinoculation) or 1.5 h at 1250×g spinning (spinoculation). After washing off unbound virus, cells were cultured in RPMI containing 10% FBS and IL-2 or 0.3% human AB serum, ITS supplement, and IL-2. HD5 was added back to cell cultures in the presence of IL-2. The level of p24 protein in culture media was measured by p24 ELISA. Data presented are the average ± standard deviation of 3 replicates. Similar results were observed in 3 independent experiments from different donors; *P<0.05, defensin-treated samples vs non-treated controls.

Spinoculation promotes HIV infection [30] by triggering dynamic actin and cofilin activity, possibly due to a cellular response to centrifugal stress [31]. Spinoculation also up-regulates HIV receptor CD4 and co-receptor CXCR4 and enhances viral binding and entry [31]. In addition to spinoculation, Furci et al culture CD4+ T cells from activated PBMCs in the presence of 0.3% human AB serum and ITS supplement, whereas we do not use spinoculation for viral infection, and we culture activated CD4+ T cells in the presence of 10% FBS, a conventional method. Note that pre-incubation of virus and defensins is performed in serum-free conditions, which is consistent in both laboratories. The presence of FBS during virus-defensin incubation significantly reduced the HIV enhancing effect in HeLa-CD4-CCR5 cells compared to HS (Table 2). HIV-1 primary isolate 20635-4 (R5 virus, clade C) [25] was diluted in serum-free RPMI and incubated with defensins at different concentrations for 1 h. Defensin-virus mixture was added to CD4+ T cells from PHA-activated PBLs (Fig. 2A) or PHA-activated CD4+ T cells (Fig. 2B) with or without spinoculation. After 2 h incubation, HIV-exposed CD4+ T cells were washed and then cultured, with or without HD5, under two different conditions: 1) in the presence of 10% FBS and IL-2, or 2) in the presence of 0.3% HS, ITS supplement and IL-2. As expected, HIV p24 levels were significantly higher in samples with spinoculation. Higher p24 levels were also observed when primary CD4+ T cells were cultured in the presence of 10% FBS than in serum-deprived conditions. It was apparent that spinoculation did not contribute to the contrasting effect of HD5. Interestingly, regardless of the method of CD4+ T cell isolation, HD5 promoted HIV replication in primary CD4+ T cells cultured in 10%FBS but inhibited viral replication in serum-deprived cells (Fig. 2). Both HIV enhancing and inhibitory effects of HD5 were dose-dependent.

**Table 2:** The effect of serum on the HIV enhancing of defensins.

Serum	HD5		HD6	
	FBS (fold increase)	HS (fold increase)	FBS (fold increase)	HS (fold increase)
0	82.9±2.6	59.0±3.7	61.2±0.5	35.2±4.1
1%	8.0±0.4	21±1.0	10.1±0.3	29.7±1.2
2%	5.1±0.5	17.8±2.9	7.7±0.1	25.3±2.8
5%	5.2±0.4	11.3±0.7	3.4±0.2	17.1±3.0
10%	2.5±0.1	7.8±0.6	2.8±0.3	13.4±3.1

Pseudotyped HIV-1_JR-FL_ luciferase reporter virus was incubated with or without defensins (5.6 µM) in the presence of FBS or human serum (HS) at various concentrations for 1 h. FBS or HS at a final concentration of 10% was added to the virus-defensin mixture before exposure to HeLa-CD4-CCR5. Luciferase activity was determined 48 h after infection. Results are expressed as fold increase compared to samples with the same concentration of FBS or HS in the absence of defensins. Assays were performed on triplicate cultures; results represent two independent experiments (mean ± SD).

**Figure 3:**
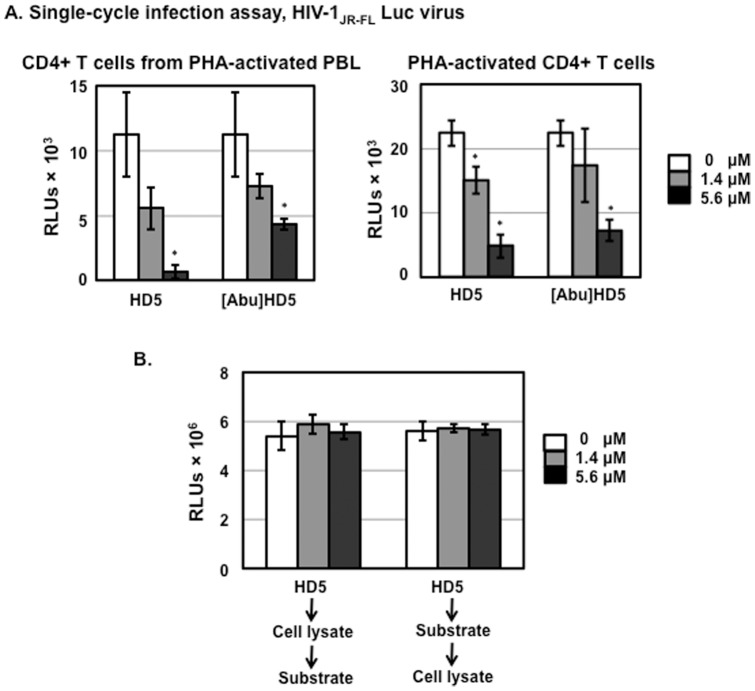
HD5 and [Abu]HD5 inhibit HIV infection in serum-deprived primary CD4+ T cells. (A). Serum-free HIV-1_JR-FL_ Env-pseudotyped reporter viruses were incubated with HD5 or its linear peptide, [Abu]HD5 for 1 h at 37°C before addition to CD4+ T cells by spinoculation. After washing off unbound virus, cells were cultured under serum-deprived condition in the presence of defensins. HIV infection was determined by measuring luciferase activity at day 3 after infection. Data presented are the average ± standard deviation of 3 replicates; *P<0.05, defensin-treated samples vs non-treated controls. Similar results were observed in 2 independent experiments. (B) To determine whether HD5 interfered with luciferase activity in vitro, 50 µl of cell lysate from HeLa-CD4-CCR5 cells or 100 µl of luciferase substrate was incubated with HD5 at different concentrations for 60 min on ice. Then, 100 µl of luciferase assay substrate or 50 µl of cell lysate was added to the mixture, respectively, and luminescence was measured.

We have previously shown that the HIV enhancing effect of HD5 requires proper folding of peptides as [Abu]HD5, the linear unstructured form of HD5, does not promote HIV infection [14], [32]. To examine whether the structure of HD5 is important for the anti-HIV activity of HD5, we assessed the effect of HD5 or [Abu]HD5 on HIV infection of CD4+ T cells negatively selected from PHA-activated PBLs or of activated CD4+ T cells under serum-deprived culture conditions using a single-cycle infection assay. The results demonstrated that, in contrast to the HIV enhancing effect of HD5, the linear form of HD5 was able to inhibit HIV infection of primary CD4+ T cells under serum-deprived conditions (Fig. 3A). The addition of human neutrophil defensin-1 (HNP-1) or rhesus theta-defensin-1 (RTD-1) at 10 µg/ml to cell lysates have been shown to inhibit luciferase activity in vitro [33]. To exclude the possibility that the anti-HIV activity of HD5 seen in Fig. 3A was due to interference with luciferase activity, defensins were incubated with either cell lysate first followed by adding to the luciferase substrate, or the luciferase substrate first followed by adding to cell lysate (Fig. 3B). Neither incubation sequence resulted in any difference compared to samples without defensins, indicating that, unlike the previous report for HNP-1 or RTD-1 [33], HD5 at either 1.4 µM (5 µg/ml) or 5.6 µM (20 µg/ml) did not interfere with the luciferase assay. Thus, the anti-HIV activity of HD5 shown in Fig. 3A was not due to defensin-mediated inhibition of luciferase activity.

### HD5-mediated HIV inhibition in serum-deprived primary CD4+ T cells is independent of HIV receptors

**Figure 4:**
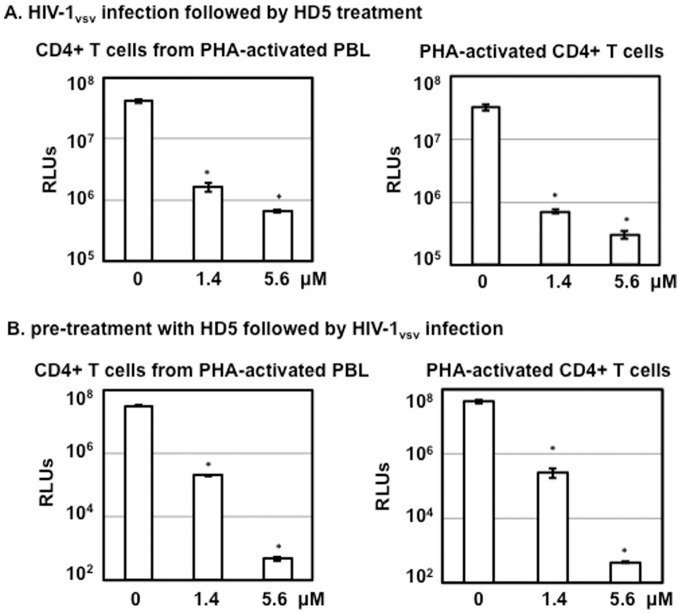
HD5-mediated inhibition of HIV in serum-deprived primary CD4+ T cells occurs independently of HIV receptors. (A) CD4+ T cells from different preparation methods were infected with pseudotyped HIV-1_VSV-G_ luciferase reporter viruses by spinoculation. Infected cells were cultured under serum-deprived conditions. HIV infection was determined by measuring luciferase activity on day 3 after infection. (B) CD4+ T cells under serum-deprived conditions were treated with HD5 at different concentrations for 1 h at 37°C followed by exposure to serum-free pseudotyped HIV-1_VSV-G_ luciferase reporter viruses by spinoculation in the presence of HD5. Cells were then cultured in RPMI containing 0.3% human AB serum, ITS supplement, IL-2, and defensins for 3 days followed by measurement of luciferase activity. Data presented are the average ± standard deviation of 3 replicates and represent 2 independent experiments. *P<0.05, defensin-treated samples vs non-treated controls.

Furci et al indicate that HD5 inhibits HIV infection by binding to both CD4 and gp120 [18]. The binding of HD5 to CD4 and gp120 is not surprising since HD5 has been shown to bind to glycosylated gp120 through its lectin-like property [34]. To examine whether the binding to CD4 and gp120 was required for the anti-HIV activity of HD5, we determined the effect of HD5 on infection by HIV-1 pseudotyped with VSV G envelope independent of HIV receptor and co-receptors for viral entry. Primary CD4+ T cells isolated by the two different methods were exposed to pseudotyped HIV-1_vsv_ luciferase reporter virus, washed, and then treated with HD5 under serum-deprived conditions (Fig. 4A). HD5 exhibited anti-HIV activity when defensin was added after viral entry (Fig. 4A). Pre-treatment of cells with defensins for 1 h followed by viral infection in the presence of HD5 also blocked HIV-1_vsv_ infection (Fig. 4B). In addition, HIV was inhibited in HD5-pretreated cells under serum-deprived conditions when defensins were not added back during infection (data not shown), suggesting that pretreatment of cells with HD5 alone was sufficient to cause HIV inhibition. These results using HIV-1vsv pseudotyped virus indicate that anti-HIV activity under serum-deprived conditions is not mediated by interference with the binding between CD4 and HIV gp120.

### HD5 induces cytotoxicity in primary CD4+ T cells under serum-deprived condition

**Figure 5:**
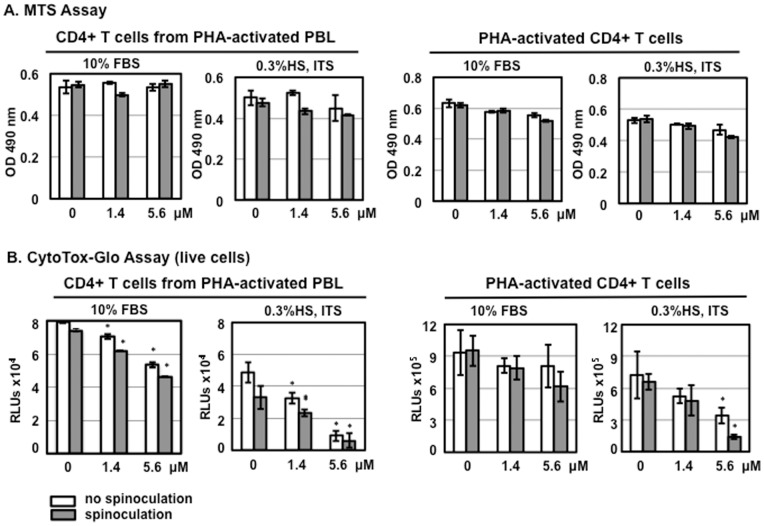
HD5 induces cytotoxicity in primary CD4+ T cells under serum-deprived conditions. CD4+ T cells (1×10^4^ cells per sample) were treated with HD5 at different concentrations with or without spinoculation. Cells were then cultured in RPMI-1640 containing10% FBS and IL-2 or RPMI-1640 containing 0.3% human AB serum, 1× ITS supplement, and IL-2. HD5 was added back. After overnight culture, cell proliferation was measured by MTS assay (A); cytotoxicity was measured by CytoTox-Glo cytotoxicity kit (B). Data presented are the average ± standard deviation of 3 replicates; *P<0.05, defensin-treated samples vs non-treated controls. Similar results were observed in 3 independent experiments.

**Figure 6:**
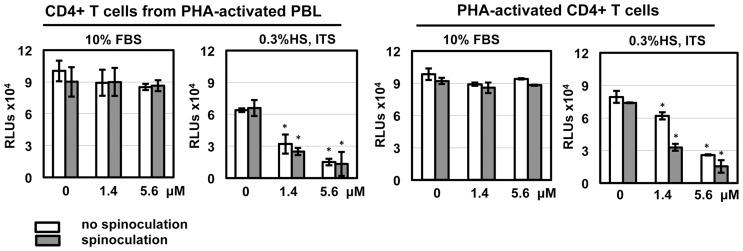
HD5 induces cytotoxicity in HIV-exposed primary CD4+ T cells under serum-deprived conditions. HIV-1_JR-FL_ was incubated with HD5 at different concentrations at 37°C for 1 h. CD4+ T cells were incubated with defensin-virus mixture with or without spinoculation as described in Fig. 2. Cells were washed and then cultured with the original concentration of HD5 in either RPMI-1640 containing10% FBS and IL-2 or RPMI-1640 containing 0.3% human AB serum, 1× ITS supplement, and IL-2. After overnight culture, cytotoxicity was measured by CytoTox-Glo cytotoxicity kit (Promega). Data presented are the average ± standard deviation of 3 replicates; *P<0.05, defensin-treated samples vs non-treated controls. Similar results were observed in 2 independent experiments.

Using the MTS assay, Furci et al. do not observe HD5-mediated cytotoxicity [18]. Although the MTS assay can be used for assaying cytotoxicity, it is primarily used to assay cell proliferation. In our experience, MTS is not as sensitive as CytoTox-Glo Cytotoxicity assay for measuring cytotoxicity in primary CD4+ T cells [35]. Nevertheless, we used both methods to determine the effect of HD5 on viability of samples using different CD4+ T cell isolation methods, spinoculation, and different culture conditions. Although higher concentrations of HD5 slightly reduced the signal of primary CD4+ T cells in the MTS assay under serum-deprived conditions after 24 h incubation, the difference was not significant compared to cells without defensins (Fig. 5A). In contrast to the results using MTS assay, in the CytoTox-Glo Cytotoxicity assay, HD5 induced significant cytotoxicity in primary CD4+ T cells under serum-deprived conditions, and this cytotoxicity was dose dependent (Fig. 5B). Overall, cell viability was reduced when CD4+ T cells were cultured in 0.3% HS, ITS, and IL-2 compared to cells cultured in 10% FBS and IL-2 regardless of isolation method. In serum-deprived cultures receiving 5.6 μM HD5, viability was reduced approximately 80% in either CD4+ T cells from PHA-activated PBLs with or without the process of spinoculation or in activated CD4+ T cells with spinoculation. As expected, 10% FBS significantly prevented cytotoxicity induced by HD5 (Fig. 5B). Similar results of HD5-induced cytotoxicity were obtained in similar experiments when HIV-exposed CD4+ T cells were assayed (Fig. 6). HD5 induced cytotoxic effects in HIV-infected CD4+ T cells under serum-deprived conditions but not in the presence of 10% FBS (Fig. 6).

**Figure 7:**
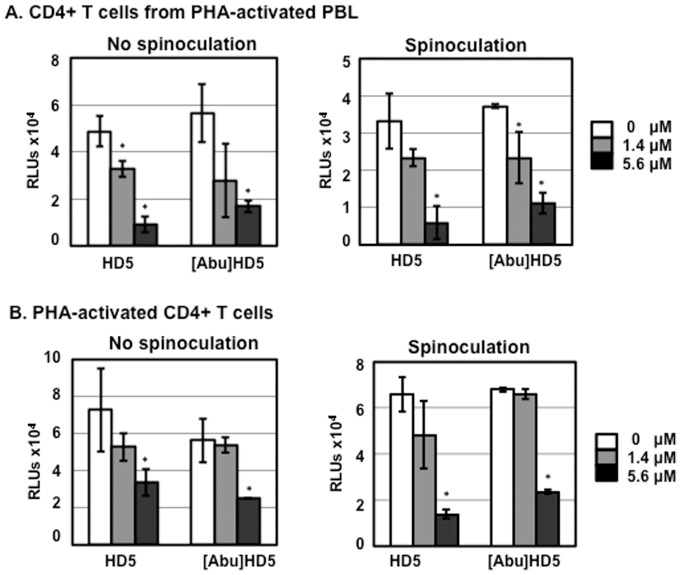
HD5 and linear peptide [Abu]HD5 induced cytotoxicity in primary CD4+ T cells under serum-deprived conditions. CD4+ T cells from PHA-activated PBLs (A) or PHA-activated CD4+ T cells (B) were exposed to HD5 or [Abu]HD5 at indicated concentrations with or without spinoculation. Cells were then cultured for 24 h under serum-deprived conditions in the presence of IL-2 and defensins. Cytotoxicity was measured by CytoTox-Glo cytotoxicity kit. Data presented are the average ± standard deviation of 3 replicates and represent 3 independent experiments. *P <0.05, defensin-treated samples vs non-treated controls.

We then examined whether a linear form of HD5, [Abu]HD5, was cytotoxic in primary CD4+ T cells under serum-deprived condition. Similar to the results of HD5-mediated HIV inhibition, linear HD5 induced cytotoxicity in CD4+ T cells from PHA-activated PBLs in a dose dependent manner with or without spinoculation (Fig. 7A). Interestingly, the cytotoxicity of linear HD5 in PHA-activated CD4+ T cells (Fig. 7B) was only observed at the higher concentration, suggesting an impact of cell purity on resistance to cytotoxicity induced by unstructured defensin.

Taken together, our results indicate that, in contrast to the HIV enhancing effect of HD5 in primary CD4+ T cells under a conventional condition (10%FBS with IL-2), anti-HIV activity and cytotoxicity of HD5 in primary CD4+ T cells under serum-deprived conditions was independent of defensin structure. Importantly, the anti-HIV activity was associated with cytotoxicity.

### HD5 induces apoptosis in primary CD4+ T cells under serum-deprived condition

**Figure 8:**
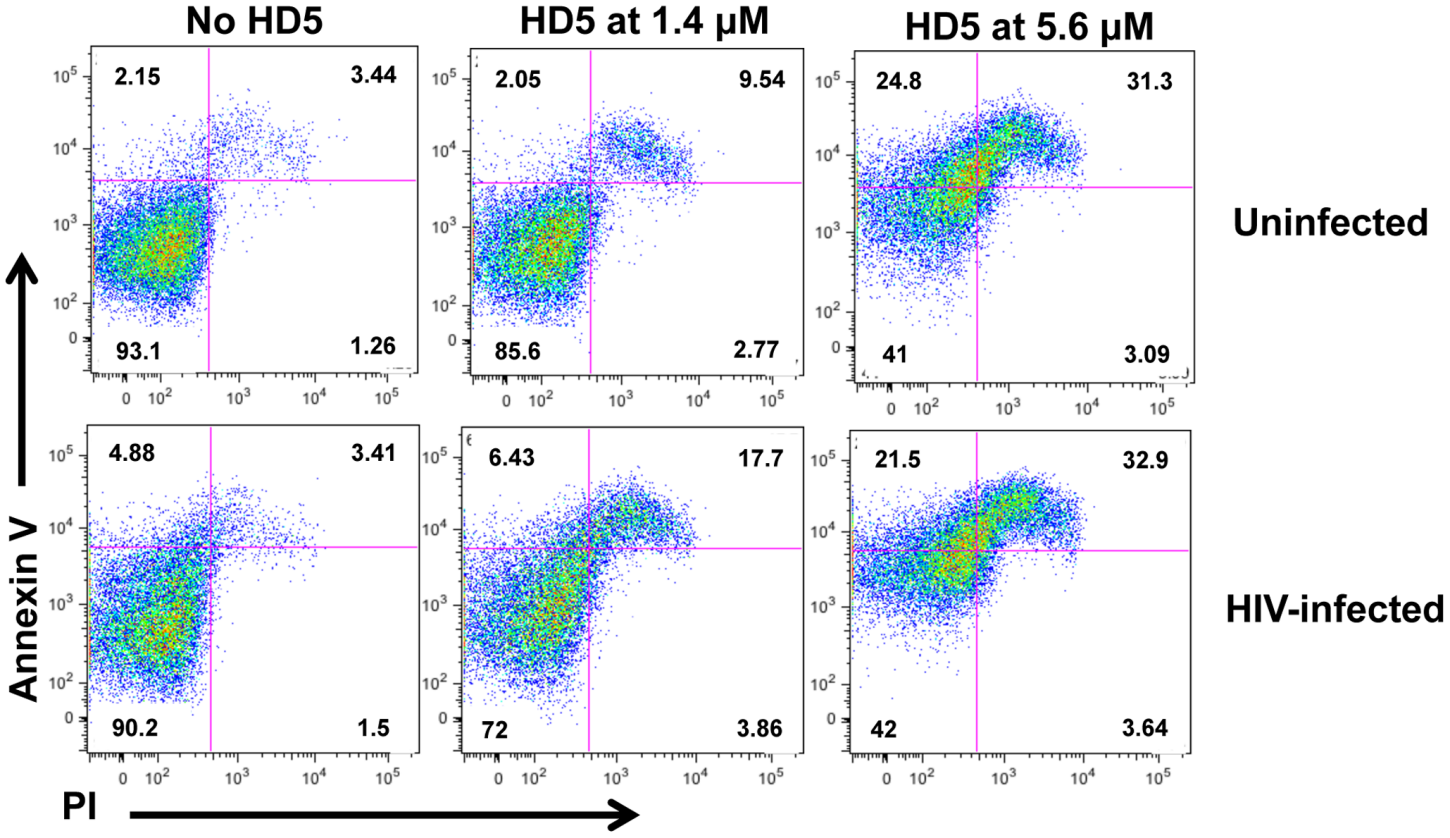
HD5 induces apoptosis in PHA-activated CD4+ T cells and HIV-infected CD4+ T cells under serum-deprived conditions. PHA-activated CD4+ T cells under serum-deprived conditions were treated with HD5 at different concentrations for 24 h before staining with Annexin V and PI. Activated CD4+ T cells were also exposed to defensin-treated HIV-1_JR-FL_ for 2 h, washed and then treated with HD5. Apoptosis was determined by FACS analysis. Similar results were obtained using cells from two independent donors.

Unlike HNP-1, a known antimicrobial peptide that induces cytotoxicity at high concentrations in the absence of serum [1], the cytotoxic effect of HD5 has not been reported previously. HNPs can induce apoptosis leading to cell death [36]. To delineate the mechanism of HD5-mediate cytotoxicity in primary CD4+ T cells under serum-deprived condition, PHA-activated CD4+ T cells were cultured in 0.3%HS, ITS, and IL-2 in the presence or absence of HD5. For comparison, the cytotoxic effect of HD5 on HIV-exposed CD4+ T cells under serum-deprived conditions was also assessed. Cells were stained with Annexin V and PI, and the apoptotic effect of HD5 was determined by FACS analysis. Cells undergoing apoptosis may translocate phosphatidylserine from their inner cytoplasmic membrane to cell surface, which can bind to Annexin V. There was an increase in Annexin V positive cells in the presence of 1.4 μM HD5 indicating HD5 induced apoptosis (Fig. 8). In the presence of 5.6 μM HD5, Annexin V positive and Annexin V/PI double positive cells, representing early and late apoptotic/necrotic cells, respectively, were significantly increased (Fig. 8). HD5-mediated apoptosis was observed as early as 2–4 h after initiation of treatment (data not shown).

### Anti-HIV activity and cytotoxicity of HD5 are not found in HeLa-CD4-CCR5 cells under serum-deprived condition

**Figure 9:**
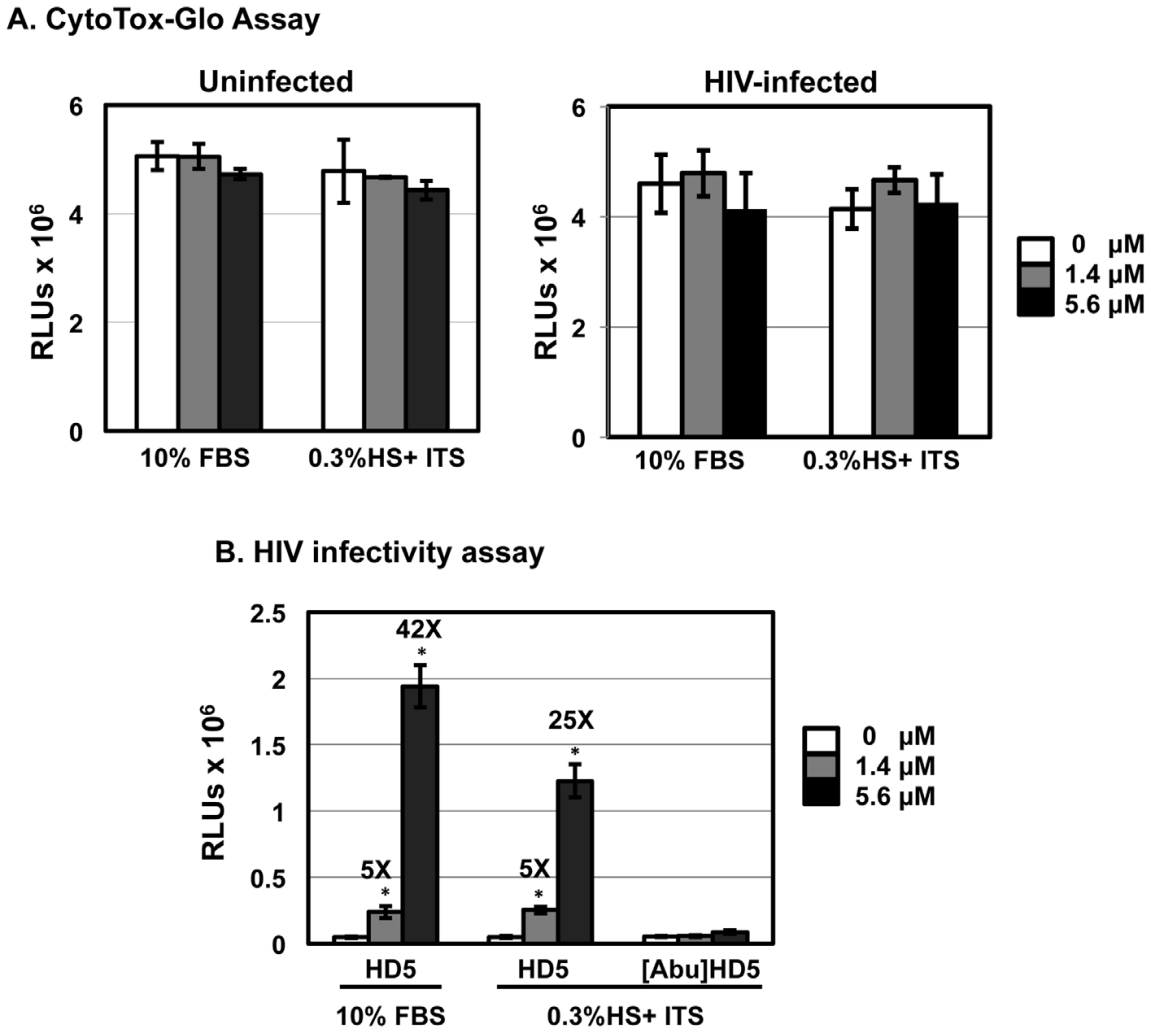
Anti-HIV activity and cytotoxicity of HD5 are not found in HeLa-CD4-CCR5 cells under serum-deprived condition. (A) HeLa-CD4-CCR5 cells were exposed to HD5 at indicated concentrations in serum-free RPMI-1640 medium for 2 h at 37°C. The effect of HD5 on HIV-exposed HeLa-CD4-CCR5 cells was also prepared as described in Fig. 6 as a comparison. The cells were then cultured for 24 h in complete medium (10%FBS) or under serum-deprived conditions. Cytotoxicity was measured by CytoTox-Glo cytotoxicity kit. (B) Serum-free HIV-1_JR-FL_ Env-pseudotyped reporter viruses were incubated with HD5 or [Abu]HD5 for 1 h at 37°C before exposure to HeLa-CD4-CCR5 cells for 2 h at 37°C. Cells were cultured in complete media or under serum-deprived conditions. Data presented are the average ± standard deviation of 3 replicates and represent 2 independent experiments. *P<0.05, defensin-treated samples vs non-treated controls.

We and others have not observed significant cytotoxicity of HD5 in either HeLa-CD4-CCR5 cells or intestinal cell lines in the absence of serum [14], [37]. To examine whether the cytotoxicity of HD5 occurred in different cell types under serum-deprived condition, HeLa-CD4-CCR5 cells were cultured in the presence of 10%FBS or 0.3% HS with ITS overnight before measurement of cytotoxicity. In addition, the effect of HD5 and linear HD5, [Abu]HD5, on HIV infection under different culture conditions was also determined. We found that HD5 was not cytotoxic to HeLa-CD4-CCR5 cells under serum-deprived conditions regardless of the presence or absence of HIV infection (Fig. 9A). The lack of cytotoxicity for HeLa-CD4-CCR5 cells was accompanied by enhanced HIV infectivity in a dose dependent manner regardless of culture conditions (Fig 9B). In contrast to the finding in primary CD4+ T cells under serum-deprived conditions, the linear HD5 had no effect on HIV infection (Fig. 9B).

## Discussion

Defensins are known to mediate complex functions in regulating immune responses and infection/transmission beyond their previously well-recognized antimicrobial properties [3], [38], [39]. The effect of defensins on viral infection is specific to the particular triad of defensin, target cell and virus [5]. While HD5 inhibits an enveloped virus such as herpes simplex virus 2 (HSV2) [40] and several non-enveloped viruses including BK virus, Human adenovirus, and papillomavirus [41], [42], [43], [44], the mechanisms of these anti-viral activities of HD5 are distinct (reviewed in [5]). In these studies including HSV2 and papillomavirus, the causative agents for sexually transmitted infections, HD5 has been incubated with virus in the absence of serum; however, the target cells were cultured in the presence of 10% FBS during the infection [40] [41], [42], [43], [44]. Using similar methods, HIV was incubated with HD5 in the absence of serum (or 1% FBS) and then CD4+ cells or HeLa-CD4-CCR5 cells were exposed to HIV in the presence of 10%FBS [14], [17], [45]. We consistently observed that HD5 promotes HIV infectivity, and this enhancing effect depends on the structure of the defensin [14], [17]. Furci et al questioned the physiological relevance of our studies when CD4+ T cells are cultured in the presence of FBS, and stated that FBS is a “non-physiological substance” [18]. In the current study, we demonstrated that the anti-HIV activity of HD5 in primary CD4+ T cells under their culture conditions was due to defensin-mediated cell death. Proteomic analysis shows that more than a thousand protein species are present in cervicovaginal fluid (reviewed in [19] [20], [21]), and there is no indication of significant cell death in endocervical CD4+ T cells from women with *Chlamydia trachomatis* infection [23], which leads to HD5 induction ([12] and our unpublished data), the CD4+ T cells cultured under serum-deprived conditions as described by Furci et al are unlikely to represent a physiologically relevant model of mucosal CD4+ T cells.

Serum deprivation alters TGF-β signaling [46], induces autophagy [47], and influences IFN-γ-mediated cell proliferation and apoptosis [48] in CD4+ T cells. In the current study, we found that under serum-deprived conditions HD5 caused apoptosis and cytotoxicity (Fig. 5, 6, 7 and 8), which was not observed in CD4+ T cells in the presence of 10%FBS. In addition to serum deprivation, a bystander effect could arise due to cell impurity. The negatively selected CD4+ T cells from PHA-activated PBMCs used by Furci et al were contaminated with CD8 cells (Fig. 1), which could secret soluble factors for cell killing or inhibiting HIV. Indeed, our results showed that HD5-induced cell death was more pronounced in CD4+ T cells from PHA-activated PBLs than in PHA-activated CD4+ T cells (Fig 5B). Spinoculation, which causes centrifugal stress, had a significant effect on HD5-induced cell death in PHA-activated CD4+ T cells (Fig. 5B, Fig. 6 and Fig. 7B). It may be that, because of the higher purity of the CD4+ T cell population in PHA-activated CD4+ T cells, in samples without spinoculation there was a reduced bystander cytotoxic effect of HD5 compared to CD4+ T cells from PHA-activated PBLs. As we expect that the cytotoxic effect of HD5 would increase over time, the lack of a significant impact of spinoculation on defensin-mediated HIV inhibition may be due to the longer incubation time of the infection assay compared to the cytotoxicity assay.

HeLa-CD4-CCR5 cells, similar to TZM-bl cells, a HeLa cell line stably transfected with CD4, CCR5, and a luciferase reporter gene under the control of HIV-1 long terminal repeat, are frequently used to assess HIV infectivity. In the absence of defensin-mediated cytotoxicity, HD5 promoted HIV infectivity of HeLa cells under serum-deprived conditions. In contrast to the anti-HIV activity of HD5 observed in serum-deprived primary CD4+ T cells that was independent of the structure of defensins, the HIV enhancing effect of HD5 in HeLa cells under serum deprived conditions required proper folding of HD5 (Fig. 9). These results suggest that the HIV enhancing effect of HD5 is structure-specific, whereas its cytotoxicity associated anti-HIV activity is independent of the structure of HD5. The underlying mechanism by which HD5 and its linear peptide induced apoptosis in primary CD4+ T cells but not HeLa cells under serum-deprived conditions remains to be determined. It is also not clear whether other positively charged peptides would induce apoptosis in serum-deprived primary CD4+ T cells.

In conclusions, we have demonstrated that the anti-HIV activity of HD5 in primary CD4+ T cells under serum-deprived conditions is a consequence of defensin-mediated cell death. The impurity of CD4+ T cells and centrifugation stress appeared to further enhance HD5-mediated cytotoxicity. In the absence of cytotoxicity, HD5 exhibited an HIV enhancing effect. Thus, results using serum-deprived primary CD4+ T cells should be interpreted with caution.

## References

[1] Ganz T (2003) Defensins: antimicrobial peptides of innate immunity. Nat Rev Immunol 3: 710–720. 12949495 10.1038/nri1180

[2] Salzman NH (2010) Paneth cell defensins and the regulation of the microbiome: detente at mucosal surfaces. Gut microbes 1: 401–406. 21468224 10.4161/gmic.1.6.14076 PMC3056107

[3] Lehrer RI, Lu W (2012) alpha-Defensins in human innate immunity. Immunological reviews 245: 84–112. 22168415 10.1111/j.1600-065X.2011.01082.x

[4] Daher KA, Selsted ME, Lehrer RI (1986) Direct inactivation of viruses by human granulocyte defensins. J Virol 60: 1068–1074. 3023659 10.1128/jvi.60.3.1068-1074.1986 PMC253347

[5] Ding J, Chou YY, Chang TL (2009) Defensins in viral infections. Journal of innate immunity 1: 413–420. 20375599 10.1159/000226256 PMC5673487

[6] Meyerholz DK, Grubor B, Gallup JM, Lehmkuhl HD, Anderson RD, et al (2004) Adenovirus-mediated gene therapy enhances parainfluenza virus 3 infection in neonatal lambs. J Clin Microbiol 42: 4780–4787. 15472341 10.1128/JCM.42.10.4780-4787.2004 PMC522350

[7] Ryan LK, Dai J, Yin Z, Megjugorac N, Uhlhorn V, et al (2011) Modulation of human {beta}-defensin-1 (hBD-1) in plasmacytoid dendritic cells (PDC), monocytes, and epithelial cells by influenza virus, Herpes simplex virus, and Sendai virus and its possible role in innate immunity. Journal of leukocyte biology 90(2): 343–356. 21551252 10.1189/jlb.0209079 PMC3133436

[8] Wehkamp J, Chu H, Shen B, Feathers RW, Kays RJ, et al (2006) Paneth cell antimicrobial peptides: topographical distribution and quantification in human gastrointestinal tissues. FEBS letters 580: 5344–5350. 16989824 10.1016/j.febslet.2006.08.083

[9] Guadalupe M, Reay E, Sankaran S, Prindiville T, Flamm J, et al (2003) Severe CD4+ T-cell depletion in gut lymphoid tissue during primary human immunodeficiency virus type 1 infection and substantial delay in restoration following highly active antiretroviral therapy. J Virol 77: 11708–11717. 14557656 10.1128/JVI.77.21.11708-11717.2003 PMC229357

[10] Jones DE, Bevins CL (1992) Paneth cells of the human small intestine express an antimicrobial peptide gene. J Biol Chem 267: 23216–23225. 1429669

[11] Quayle AJ, Porter EM, Nussbaum AA, Wang YM, Brabec C, et al (1998) Gene expression, immunolocalization, and secretion of human defensin-5 in human female reproductive tract. Am J Pathol 152: 1247–1258. 9588893 PMC1858596

[12] Porter E, Yang H, Yavagal S, Preza GC, Murillo O, et al (2005) Distinct defensin profiles in Neisseria gonorrhoeae and Chlamydia trachomatis urethritis reveal novel epithelial cell-neutrophil interactions. Infect Immun 73: 4823–4833. 16040996 10.1128/IAI.73.8.4823-4833.2005 PMC1201278

[13] Fan SR, Liu XP, Liao QP (2008) Human defensins and cytokines in vaginal lavage fluid of women with bacterial vaginosis. Int J Gynaecol Obstet 103(1): 50–54. 18635180 10.1016/j.ijgo.2008.05.020

[14] Klotman ME, Rapista A, Teleshova N, Micsenyi A, Jarvis GA, et al (2008) Neisseria gonorrhoeae-Induced Human Defensins 5 and 6 Increase HIV Infectivity: Role in Enhanced Transmission. J Immunol 180: 6176–6185. 18424739 10.4049/jimmunol.180.9.6176 PMC3042429

[15] Zhu T, Mo H, Wang N, Nam DS, Cao Y, et al (1993) Genotypic and phenotypic characterization of HIV-1 patients with primary infection. Science 261: 1179–1181. 8356453 10.1126/science.8356453

[16] van't Wout AB, Kootstra NA, Mulder-Kampinga GA, Albrecht-van Lent N, Scherpbier HJ, et al (1994) Macrophage-tropic variants initiate human immunodeficiency virus type 1 infection after sexual, parenteral, and vertical transmission. The Journal of clinical investigation 94: 2060–2067. 7962552 10.1172/JCI117560 PMC294642

[17] Rapista A, Ding J, Benito B, Lo YT, Neiditch MB, et al (2011) Human defensins 5 and 6 enhance HIV-1 infectivity through promoting HIV attachment. Retrovirology 8: 45. 21672195 10.1186/1742-4690-8-45 PMC3146398

[18] Furci L, Tolazzi M, Sironi F, Vassena L, Lusso P (2012) Inhibition of HIV-1 infection by human alpha-defensin-5, a natural antimicrobial peptide expressed in the genital and intestinal mucosae. PloS one 7: e45208. 23028850 10.1371/journal.pone.0045208 PMC3459904

[19] Zegels G, Van Raemdonck GA, Tjalma WA, Van Ostade XW (2010) Use of cervicovaginal fluid for the identification of biomarkers for pathologies of the female genital tract. Proteome science 8: 63. 21143851 10.1186/1477-5956-8-63 PMC3016264

[20] Shaw JL, Smith CR, Diamandis EP (2007) Proteomic analysis of human cervico-vaginal fluid. Journal of proteome research 6: 2859–2865. 17567164 10.1021/pr0701658

[21] Zegels G, Van Raemdonck GA, Coen EP, Tjalma WA, Van Ostade XW (2009) Comprehensive proteomic analysis of human cervical-vaginal fluid using colposcopy samples. Proteome science 7: 17. 19374746 10.1186/1477-5956-7-17 PMC2678104

[22] Venkataraman N, Cole AL, Svoboda P, Pohl J, Cole AM (2005) Cationic polypeptides are required for anti-HIV-1 activity of human vaginal fluid. J Immunol 175: 7560–7567. 16301665 10.4049/jimmunol.175.11.7560

[23] Ficarra M, Ibana JS, Poretta C, Ma L, Myers L, et al (2008) A distinct cellular profile is seen in the human endocervix during Chlamydia trachomatis infection. Am J Reprod Immunol 60: 415–425. 18798835 10.1111/j.1600-0897.2008.00639.x PMC2574558

[24] Wu Z, Ericksen B, Tucker K, Lubkowski J, Lu W (2004) Synthesis and characterization of human alpha-defensins 4-6. The journal of peptide research: official journal of the American Peptide Society 64: 118–125. 15317502 10.1111/j.1399-3011.2004.00179.x

[25] Brown BK, Darden JM, Tovanabutra S, Oblander T, Frost J, et al (2005) Biologic and genetic characterization of a panel of 60 human immunodeficiency virus type 1 isolates, representing clades A, B, C, D, CRF01_AE, and CRF02_AG, for the development and assessment of candidate vaccines. Journal of virology 79: 6089–6101. 15857994 10.1128/JVI.79.10.6089-6101.2005 PMC1091694

[26] Chen BK, Saksela K, Andino R, Baltimore D (1994) Distinct modes of human immunodeficiency virus type 1 proviral latency revealed by superinfection of nonproductively infected cell lines with recombinant luciferase-encoding viruses. J Virol 68: 654–660. 7507183 10.1128/jvi.68.2.654-660.1994 PMC236499

[27] Connor RI, Sheridan KE, Ceradini D, Choe S, Landau NR (1997) Change in coreceptor use coreceptor use correlates with disease progression in HIV-1–infected individuals. J Exp Med 185: 621–628. 9034141 10.1084/jem.185.4.621 PMC2196142

[28] Chang TL, Vargas J Jr, DelPortillo A, Klotman ME (2005) Dual role of alpha-defensin-1 in anti-HIV-1 innate immunity. J Clin Invest 115: 765–773. 15719067 10.1172/JCI200521948 PMC548697

[29] Martinez-Lorenzo MJ, Anel A, Gamen S, Monle NL, Lasierra P, et al (1999) Activated human T cells release bioactive Fas ligand and APO2 ligand in microvesicles. Journal of immunology 163: 1274–1281. 10415024

[30] O'Doherty U, Swiggard WJ, Malim MH (2000) Human immunodeficiency virus type 1 spinoculation enhances infection through virus binding. Journal of virology 74: 10074–10080. 11024136 10.1128/jvi.74.21.10074-10080.2000 PMC102046

[31] Guo J, Wang W, Yu D, Wu Y (2011) Spinoculation triggers dynamic actin and cofilin activity that facilitates HIV-1 infection of transformed and resting CD4 T cells. Journal of virology 85: 9824–9833. 21795326 10.1128/JVI.05170-11 PMC3196392

[32] Rapista A, Ding J, Benito B, Lo YT, Neiditch MB, et al (2011) Human defensins 5 and 6 enhance HIV-1 infectivity through promoting HIV attachment. Retrovirology 8: 45. 21672195 10.1186/1742-4690-8-45 PMC3146398

[33] Seidel A, Ye Y, de Armas LR, Soto M, Yarosh W, et al (2010) Cyclic and acyclic defensins inhibit human immunodeficiency virus type-1 replication by different mechanisms. PloS one 5: e9737. 20305815 10.1371/journal.pone.0009737 PMC2840026

[34] Lehrer RI, Jung G, Ruchala P, Andre S, Gabius HJ, et al (2009) Multivalent binding of carbohydrates by the human {alpha}-defensin, HD5. J Immunol 183: 480–490. 19542459 10.4049/jimmunol.0900244

[35] Ding J, Chang TL (2012) TLR2 activation enhances HIV nuclear import and infection through T cell activation-independent and -dependent pathways. Journal of immunology 188: 992–1001. 10.4049/jimmunol.1102098 PMC3262879 22210918

[36] Aarbiou J, Tjabringa GS, Verhoosel RM, Ninaber DK, White SR, et al (2006) Mechanisms of cell death induced by the neutrophil antimicrobial peptides alpha-defensins and LL-37. Inflammation research: official journal of the European Histamine Research Society [et al] 55: 119–127. 10.1007/s00011-005-0062-9 16673155

[37] Porter EM, Liu L, Oren A, Anton PA, Ganz T (1997) Localization of human intestinal defensin 5 in Paneth cell granules. Infect Immun 65: 2389–2395. 9169779 10.1128/iai.65.6.2389-2395.1997 PMC175331

[38] Yang D, Oppenheim JJ (2004) Antimicrobial proteins act as “alarmins” in joint immune defense. Arthritis and rheumatism 50: 3401–3403. 15529365 10.1002/art.20604

[39] Rehaume LM, Hancock RE (2008) Neutrophil-derived defensins as modulators of innate immune function. Crit Rev Immunol 28: 185–200. 19024344 10.1615/critrevimmunol.v28.i3.10

[40] Hazrati E, Galen B, Lu W, Wang W, Ouyang Y, et al (2006) Human alpha- and beta-defensins block multiple steps in herpes simplex virus infection. J Immunol 177: 8658–8666. 17142766 10.4049/jimmunol.177.12.8658

[41] Dugan AS, Maginnis MS, Jordan JA, Gasparovic ML, Manley K, et al (2008) Human alpha-defensins inhibit BK virus infection by aggregating virions and blocking binding to host cells. J Biol Chem 283: 31125–31132. 18782756 10.1074/jbc.M805902200 PMC2576552

[42] Smith JG, Nemerow GR (2008) Mechanism of adenovirus neutralization by Human alpha-defensins. Cell Host Microbe 3: 11–19. 18191790 10.1016/j.chom.2007.12.001

[43] Buck CB, Day PM, Thompson CD, Lubkowski J, Lu W, et al (2006) Human {alpha}-defensins block papillomavirus infection. Proc Natl Acad Sci U S A 103: 1516–1521. 16432216 10.1073/pnas.0508033103 PMC1360544

[44] Gounder AP, Wiens ME, Wilson SS, Lu W, Smith JG (2012) Critical determinants of human alpha-defensin 5 activity against non-enveloped viruses. The Journal of biological chemistry 287: 24554–24562. 22637473 10.1074/jbc.M112.354068 PMC3397880

[45] Ding J, Rapista A, Teleshova N, Lu W, Klotman ME, et al (2011) Mucosal human defensins 5 and 6 antagonize the anti-HIV activity of candidate polyanion microbicides. Journal of innate immunity 3: 208–212. 21160168 10.1159/000322355 PMC3072205

[46] Classen S, Zander T, Eggle D, Chemnitz JM, Brors B, et al (2007) Human resting CD4+ T cells are constitutively inhibited by TGF beta under steady-state conditions. Journal of immunology 178: 6931–6940. 10.4049/jimmunol.178.11.6931 17513742

[47] Li C, Capan E, Zhao Y, Zhao J, Stolz D, et al (2006) Autophagy is induced in CD4+ T cells and important for the growth factor-withdrawal cell death. Journal of immunology 177: 5163–5168. 10.4049/jimmunol.177.8.5163 17015701

[48] Novelli F, Di Pierro F, Francia di Celle P, Bertini S, Affaticati P, et al (1994) Environmental signals influencing expression of the IFN-gamma receptor on human T cells control whether IFN-gamma promotes proliferation or apoptosis. Journal of immunology 152: 496–504. 8283033

